# Normalization of HE-stained histological images using cycle consistent generative adversarial networks

**DOI:** 10.1186/s13000-021-01126-y

**Published:** 2021-08-06

**Authors:** Marlen Runz, Daniel Rusche, Stefan Schmidt, Martin R. Weihrauch, Jürgen Hesser, Cleo-Aron Weis

**Affiliations:** 1 Institute of Pathology, University Medical Centre Mannheim, Heidelberg University, Mannheim, Germany; 2 Mannheim Institute for Intelligent Systems in Medicine (MIISM), Medical Faculty Mannheim, Heidelberg University, Mannheim, Germany; 3 Center for Mass Spectrometry and Optical Spectroscopy (CeMOS), Mannheim University of Applied Sciences, Mannheim, Germany; 4 Smart In Media AG, Köln, Germany; 5 Interdisciplinary Center for Scientific Computing (IWR), Heidelberg University, Heidelberg, Germany; 6 Central Institute for Computer Engineering (ZITI), Heidelberg University, Heidelberg, Germany

**Keywords:** Histology stain normalization, HE-stain, Digital pathology, Generative adversarial networks, Unpaired image-to-image translation, Style transfer, Deep learning

## Abstract

**Background:**

Histological images show strong variance (e.g. illumination, color, staining quality) due to differences in image acquisition, tissue processing, staining, etc. This can impede downstream image analysis such as staining intensity evaluation or classification. Methods to reduce these variances are called image normalization techniques.

**Methods:**

In this paper, we investigate the potential of CycleGAN (cycle consistent Generative Adversarial Network) for color normalization in hematoxylin-eosin stained histological images using daily clinical data with consideration of the variability of internal staining protocol variations. The network consists of a generator network *G*_*B*_ that learns to map an image *X* from a source domain *A* to a target domain *B*, i.e. *G*_*B*_:*X*_*A*_→*X*_*B*_. In addition, a discriminator network *D*_*B*_ is trained to distinguish whether an image from domain *B* is real or generated. The same process is applied to another generator-discriminator pair (*G*_*A*_,*D*_*A*_), for the inverse mapping *G*_*A*_:*X*_*B*_→*X*_*A*_. Cycle consistency ensures that a generated image is close to its original when being mapped backwards (*G*_*A*_(*G*_*B*_(*X*_*A*_))≈*X*_*A*_ and vice versa). We validate the CycleGAN approach on a breast cancer challenge and a follicular thyroid carcinoma data set for various stain variations. We evaluate the quality of the generated images compared to the original images using similarity measures. In addition, we apply stain normalization on pathological lymph node data from our institute and test the gain from normalization on a ResNet classifier pre-trained on the Camelyon16 data set.

**Results:**

Qualitative results of the images generated by our network are compared to original color distributions. Our evaluation indicates that by mapping images to a target domain, the similarity training images from that domain improves up to 96%. We also achieve a high cycle consistency for the generator networks by obtaining similarity indices greater than 0.9. When applying the CycleGAN normalization to HE-stain images from our institute the kappa-value of the ResNet-model that is only trained on Camelyon16 data is increased more than 50%.

**Conclusions:**

CycleGANs have proven to efficiently normalize HE-stained images. The approach compensates for deviations resulting from image acquisition (e.g. different scanning devices) as well as from tissue staining (e.g. different staining protocols), and thus overcomes the staining variations in images from various institutions.The code is publicly available at https://github.com/m4ln/stainTransfer_CycleGAN_pytorch. The data set supporting the solutions is available at 10.11588/data/8LKEZF.

## Background

In both histology and surgical pathology, the inherent individual appearance of the considered object on the one hand or the different staining protocols on the other hand must be compensated in addition to factors that influence the image acquisition (e.g scanning devices). This demand applies to hematoxylin-eosin (HE) staining being the standard method in pathology but also to all other histochemical and immunohistochemical staining. Regarding HE-staining, solutions and protocols are standardized at first glance. However, even within a single institution, protocols may vary slightly and may not be coordinated with other institutes. Especially when training deep neural networks, for example for image classification, there is a need for stain normalization of images so that models are transferable to other data sets.

The term color normalization is an umbrella term for image processing techniques compensating for effects such as variable illumination, camera setting, etc. This evident request drives an active research. Conventional image processing such as color deconvolution or look-up tables with the need for selecting a reference template slide for normalization are widespread [1–6]. A particular but quite similar issue is stain quantification [7, 8]. Recent publications investigated in the use of deep learning approaches with Generative Adversarial Networks (GANs) and showed the benefits compared to the conventional methods [9, 10]. It was also shown how normalizing images using GANs can highly improve results of image classification [11] or segmentation [12]. Mahapatra et al. [13] integrate self-supervised semantic information such as geometric and structural patterns at different layers to improve stain normalization with CycleGANs.

In this work, we investigate the potential and limitation of a machine learning-based approach for normalization with a cycle consistent Generative Adversarial Network (CycleGAN) which learns the mapping from one HE-stain variant to an other. The approach we follow was proposed by Zhu et al. [14]. An image-to-image mapping is learned between two different HE-stained data sets to generate fake images in each image domain. We apply the technique to two independent data sets: the Mitosis-Atypia-14 challenge which provides two image sets of breast cancer tissue scanned with two different devices, and our *HE-Staining Variation* (HEV) data set, showing follicular thyroid carcinoma slices stained with different protocols. We evaluate the results using the Fréchet Inception Distance (FID) and the Structural Similarity Index Measure (SSIM). In addition, we apply our method to a tumor classification task of lymph node samples, in which we apply stain normalization on pathological lymph node data from our institute and test the gain from normalization on a ResNet classifier pre-trained on the Camelyon16 data set. We show that normalization can increase the confidence of the classifier not only prior to the training, but also when the test data is normalized on the training set and applied to the model after training. In addition to that, we demonstrate the potential of using this normalization for recoloring images with stains that are not present in the original data; and at the same time we achieve a higher accuracy in our classification task.

We provide both, our implementation at https://github.com/m4ln/stainTransfer_CycleGAN_pytorch, and our data set for normalization and classification at 10.11588/data/8LKEZF.

## Methods

### CycleGAN formulation

The CycleGAN from Zhu et al. [14] consists of two generator and discriminator pairs each of which learns the mapping from one image domain to the other. Given the image domains *A* and *B* with training images *X*_*A*_ and *X*_*B*_, the generator *G*_*B*_ learns the mapping from *A* to *B* such that *G*_*B*_:*X*_*A*_→*X*_*B*_, while the generator *G*_*A*_ learns the mapping in reverse direction, i.e. *G*_*A*_:*X*_*B*_→*X*_*A*_. A discriminator *D* is a binary classifier. It decides whether a sample is real (1), i.e. given from the training data set, or fake (0), i.e. produced by the generator. More precisely, discriminator *D*_*B*_ learns to distinguish between real images $X_{B}^{real}$ and generated ones $X_{B}^{fake}$, while in the same way, *D*_*A*_ is trained to discriminate between $X_{A}^{real}$ and $X_{A}^{fake}$.

For training, the objective function to be optimized is modeled by two loss functions: the adversarial loss $\mathcal {L}^{adv}$ [15] and the cycle consistency loss $\mathcal {L}^{cyc}$ [14].

#### Adversarial loss

Introduced by Goodfellow et al. [15] the adversarial loss refers to the two-player game between the generator and the discriminator networks. More precisely, for the mapping *G*_*B*_:*X*_*A*_→*X*_*B*_, the discriminator *D*_*B*_ is trained to classify $X_{B}^{real}$ and $X_{B}^{fake}$ correctly, while the generator seeks $X_{B}^{fake}$ being classified as real by the discriminator. In this way, both, the generator and the discriminator try to fool the other. Zhu et al. [14] use the least-squares loss as objective since it ensures stability during training and generates higher quality results. Thus, the adversarial loss function is expressed as follows [14]:

$$\begin{aligned} {}\min_{G_{B}}\max_{D_{B}}\mathcal{L}_{B}^{adv} &= \mathbb{E}_{X_{B}^{real}} \left[ D_{B} \left(X_{B}^{real} \right)^{2} \right]\\ &\quad+ \mathbb{E}_{X_{A}^{real}} \left[ \left(D_{B} \left(G_{B} \left(X_{A}^{real}\right) \right)-1 \right)^{2} \right], \end{aligned} $$

with $\mathbb {E}$ being the expected value over all instances of $X_{A}^{real}$ and $X_{B}^{real}$. In the same way, we can formulate the adversarial loss for the inverse mapping function *G*_*A*_:*X*_*B*_→*X*_*A*_, i.e.

$$\begin{aligned} {}\min_{G_{A}}\max_{D_{A}}\mathcal{L}_{A}^{adv}&= \mathbb{E}_{X_{A}^{real}} \left[ D_{A} \left(X_{A}^{real} \right)^{2} \right]\\ &\quad+ \mathbb{E}_{X_{B}^{real}} \left[ \left(D_{A} \left(G_{A} \left(X_{B}^{real}\right) \right)-1 \right)^{2} \right], \end{aligned} $$

Thus, the total adversarial loss $\mathcal {L}^{adv}$ is obtained by the sum of both terms $\mathcal {L}^{adv}_{A}$ and $\mathcal {L}^{adv}_{B}$.

#### Cycle consistency loss

Zhu et al. [14] presented this loss function to enforce that both mapping functions *G*_*A*_ and *G*_*B*_ learned by the generators are inverse functions. In other words, if an image is mapped from one domain to the other domain the backward mapping should bring the image back to its original state. Thus, it must satisfy the cycle $X_{A}^{real} \rightarrow G_{B}(X_{A}^{real}) \rightarrow G_{A}(G_{B}(X_{A}^{real})) = X_{A}^{rec} \approx X_{A}^{real}$ and in the same way for $X_{B}^{real} \rightarrow G_{A}(X_{B}^{real}) \rightarrow G_{B}(G_{A}(X_{B}^{real})) = X_{B}^{rec} \approx X_{B}^{real}$ for the backward mapping. Therefore, the total cycle consistency is given by:

$$\begin{aligned} {}\mathcal{L}^{cyc}&= \underbrace{\mathbb{E}_{X_{A}^{real}} \left[ \left\| G_{A} \left(G_{B} \left(X_{A}^{real}\right) \right) - X_{A}^{real} \right\|_{1} \right]}_{\mathcal{L}^{cyc}_{A}}\\ &\quad+ \underbrace{\mathbb{E}_{X_{B}^{real}} \left[ \left\| G_{B} \left(G_{A} \left(X_{B}^{real}\right) \right) - X_{B}^{real} \right\|_{1} \right]}_{\mathcal{L}^{cyc}_{B}}, \end{aligned} $$

where ∥·∥_1_ denotes the *ℓ*_1_-Norm.

Hence, the total loss function is

$$\underset{G_{B}, G_{A}}{\arg\min} \ \underset{D_{B}, D_{A}}{\arg\max} \ \mathcal{L} = \mathcal{L}^{adv} + \lambda \mathcal{L}^{cyc}, $$

with *λ* being a regularization factor to control the relative importance of both, adversarial and cycle consistency losses.

**Fig. 1:**
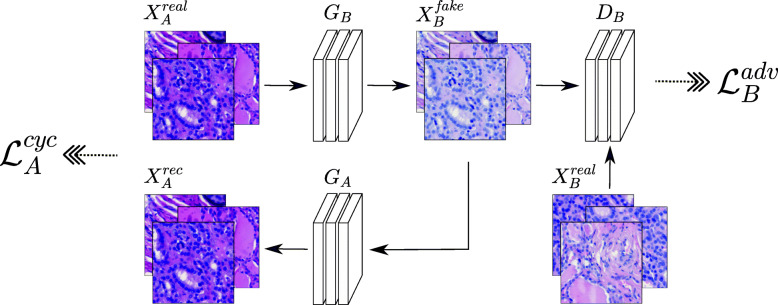
Illustration of the applied CycleGAN architecture for mapping images from domain *A* to domain *B*. A real sample image $X_{A}^{real}$ is mapped to domain *B* by the generator $G_{B}: X_{A}^{real} \rightarrow X_{B}^{fake}$ and then back to domain *A* by the generator $G_{A}: X_{B}^{fake} \rightarrow X_{A}^{rec}$. The discriminator *D*_*B*_ differentiates between the generated image $X_{B}^{fake}$ and a real sample image $X_{B}^{real}$. The same process is done for the reverse direction when mapping a real sample image $X_{B}^{real}$ from domain *B* to domain *A* and backwards, i.e $X_{B} \xrightarrow {G_{A}} X_{A}^{fake} \xrightarrow {G_{B}} X_{B}^{rec}$. During training, the loss is computed by the adversarial loss $\mathcal {L}^{adv}$ and the cycle consistency loss $\mathcal {L}^{cyc}$

Figure 1 illustrates the CycleGAN structure for mapping an image from domain *A* to domain *B* by $G_{B}: X_{A}^{real}\rightarrow X_{B}^{fake}$ and backwards by $G_{A}: X_{B}^{fake}\rightarrow X_{A}^{rec}$. The discriminator *D*_*B*_ tries to identify if an image is generated $X_{B}^{fake}$ or real $X_{B}^{real}$. During training, the network is optimized by computing the adversarial loss $\mathcal {L}^{adv}$ and the cycle consistency loss $\mathcal {L}^{cyc}$. The same process is done for the reverse direction when a real sample image $X_{B}^{real}$ is mapped from domain *B* to domain *A*, i.e $X_{B}^{real} \xrightarrow {G_{A}} X_{A}^{fake} \xrightarrow {G_{B}} X_{B}^{rec}$.

### Data sets

Two data sets are used to asses the quality of stain normalization using CycleGAN: **(a)** The Mitos-Atypia-14 challenge data set in which the HE-stain in images appears different in color and resolution due to different scanning devices. **(b)** Our clinical HE-Staining Variation (HEV) data set, which contains images of serial sections that were subjected to different staining protocols. In addition, we apply our CycleGAN normalization method in a tumor lymph node classification task trained on the Camelyon16 data set [16] and tested on the TumorLymphnode data set collected at our institute.

#### Mitos-Atypia-14

This is a publicly available challenge data set containing breast-cancer images [17]. The tissue was HE-stained and scanned by two different whole-slide image (WSI) scanners: the Aperio ScanScope XT and the Hamamatsu Nanozoomer 2.0-HT. Both devices scan images with different resolutions, the Aperio 1539×1376 pixels and the Hamamatsu 1663×1485 pixels at X20 and X40 magnification. From each scanned set, 7,936 tiles are selected for training and 15,000 tiles for testing. We resize the images to 1024×1024 pixels and extract image tiles of 256×256 pixels as input to our network.

#### HE-Staining variation

**Fig. 2:**
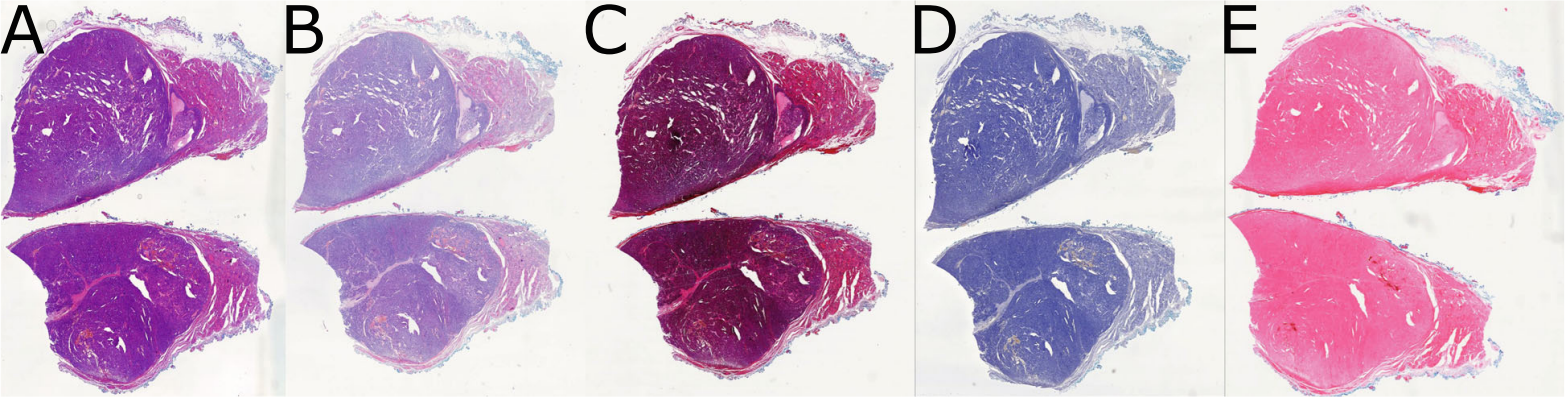
Exemplary miniature image of the WSI that forms the HEV data set. Serial tissue sections from a thyroid tissue with a follicular carcinoma with HE-staining. For every slide the staining protocol is intentionally modified: **A**) Standard protocol at the Institute of Pathology, Medical Faculty Mannheim, Heidelberg University (*HE*) **B**) Shortened staining time (*shortHE*) **C**) Prolonged staining time (*longHE*) **D**) Only hematoxylin-stain (*onlyH*) **E**) Only eosin-stain (*onlyE*)

At the Institute of Pathology, Medical Faculty Mannheim, Heidelberg University we collected serial sections of a follicular thyroid carcinoma and stained them with the following HE-staining variants: standard protocol (of the Institute of Pathology, Mannheim) HE-stain (henceforth *HE*), intentionally stained too short (henceforth *shortHE*), intentionally stained too long (henceforth *longHE*), only stained with hematoxylin (henceforth *onlyH*), and only stained with Eosin (henceforth *onlyE*). Figure 2 shows thumbnails from each WSI. For each set, we extract tiles of 256×256 pixels. We collect 10,000 and 15,000 tiles for training and testing, respectively. The whole data set including our training patches is made publicly available [18].

#### Image classification

The effect of HE-staining normalization is applied on a tumor classification task using two sets of data: **(1)** The Camelyon16 data set [16] containing the classes normal lymph node (*n*=3,318) and carcinoma infiltration (*n*=3,591), **(2)** Slides with normal lymph nodes and carcinoma infiltration from an interval of 15a (hereinafter called TumorLymphnode or short TL data set) collected from the Institute of Pathology, Heidelberg University. These are segmented manually and then cropped into tiles (*n*=3605 with normal lymph node parts, *n*=3,600 with carcinoma infiltration).

### Training details

#### CycleGAN

In the first setting, we train our model on the Mitos-Atypia-14 challenge to learn the mapping between the two image sets *X*_*A*_ and *X*_*B*_ obtained by the scanners Aperio and Hamamatsu, respectively. We then train four additional models on the HEV data set, with set *A* being the standard stained tissue (see Fig. 2**A**) and set *B* being one of the other stained tissues (see Fig. 2**B-E**). For each model we choose 10,000 images for training. An overview of the experiments on stain normalization are given in the Appendix.

For the classification task, we train a CycleGAN to normalize the Camelyon16 data set to the standard HE and hematoxylin staining of the HEV data set and to the TL data set. In the same way we train another model to normalize the TL data set to the Cameylon16 data set and to the HE and hematoxylin staining of the HEV data set. For each image set we choose 5,000 images for training.

For all models we use the same network architecture as described by Zhu et al. [14]. We train each network for 60 epochs in total where the initial learning-rate is set to 2*e*^−4^ and then decreases to zero after every 30 epochs. The regularization factor *λ* is set to 10 for all experiments. Adam optimizer is used (*β*_1_=0.5,*β*_2_=0.999) with a batch size of 1. We train and evaluate the models on an NVIDIA Quadro P6000 graphics card.

#### ResNet

A ResNet [19] - in its Pytorch-implementation - is trained on the original Camelyon16 data set and its normalized versions to the HEV and TL data sets (using CycleGAN), to classify images into the two classes normal lymph node and carcinoma infiltration. Each network is trained for 100 epochs in total with a learning-rate of 2*e*^−4^.

### Evaluation metrics

To assess the quality of the images generated by the CycleGAN we use the Fréchet Inception Distance (FID), to compare the distribution of generated images with the distribution of real images. In addition, the successful training of both generators is assessed by computing the Structural Similarity Index Measure (SSIM) in various windows between a real image and its reconstruction through the generator. For the classification task, we calculate kappa-values from the confusion matrix.

#### FID

This metric consists of the Fréchet distance also known as Wasserstein-2 distance computed on the basis of feature vectors. Here, a feature vector is the 2048-sized output of a pre-trained inception v3 model applied on one image. For the whole set of input images we get a sample of feature vectors with *m*_1_ as its collective mean and *C*_1_ as its covariance while for the GAN output images we get *m*_2_,*C*_2_ respectively [20]. The Fréchet distance is then applied to calculate the minimum distance between the means and covariances [21]:

$$\begin{aligned} {}d^{2}\ \left((m_{1}, C_{1}),(m_{2}, C_{2}) \right)&= ||m_{1} - m_{2}||^{2}\ \\ &\quad+ Tr\left(C_{1} + C_{2} - 2 \sqrt{C_{1} {\ast} C_{2}}\right). \end{aligned} $$

For identical image sets the FID is zero, whereas it increases with noise and disturbances. The python code for computing FID scores is used from [22].

#### SSIM

For a given original image *x* and the corresponding output of the GAN *y* the features luminance *l*(*x*,*y*), contrast *c*(*x*,*y*) and structure *s*(*x*,*y*) are compared on basis of the respective average, variance and covariance. The product of these components with the weighting factors *α*,*β*,*γ* yields the SSIM:

$${\mathrm SSIM}(\mathbf{x},\mathbf{y})=\left[l(\mathbf{x},\mathbf{y})\right]^{\alpha}\cdot\left[c(\mathbf{x},\mathbf{y})\right]^{\beta}\cdot\left[s(\mathbf{x},\mathbf{y})\right]^{\gamma}. $$

The SSIM metric is applied on image pairs showing the same image content. It ranges from 0 to 1 and equals one only for exact identical images. An SSIM close to zero hardly represents similar images [23]. We use the python code from [24] to compute SSIM scores.

#### Cohen’s kappa

The kappa-values are calculated from the confusion matrix using scikit-learn’s function cohen_kappa_score [25]. The kappa score ranges from -1 and 1. A value ≥ 0.8 is considered as almost perfect agreement while ≤ 0 means no agreement (i.e. random labels) [26].

## Results

We show the visual and evaluation results of our CycleGAN experiments on the Mitos-Atypia-14 and HEV data sets. For generators *G*_*A*_ and *G*_*B*_, image tiles from image domains *A* and *B* can be normalized in both directions such that $X_{A}^{real}\xrightarrow {G_{B}} X_{B}^{fake} \xrightarrow {G_{A}} X_{A}^{rec}$ and $X_{B}^{real} \xrightarrow {G_{A}} X_{A}^{fake} \xrightarrow {G_{B}} X_{B}^{rec} $. In addition, we show the results of our experiments on the lymph node classification task with HE-stain normalization.

### CycleGAN visual results

#### Mitos-Atypia-14

**Fig. 3:**
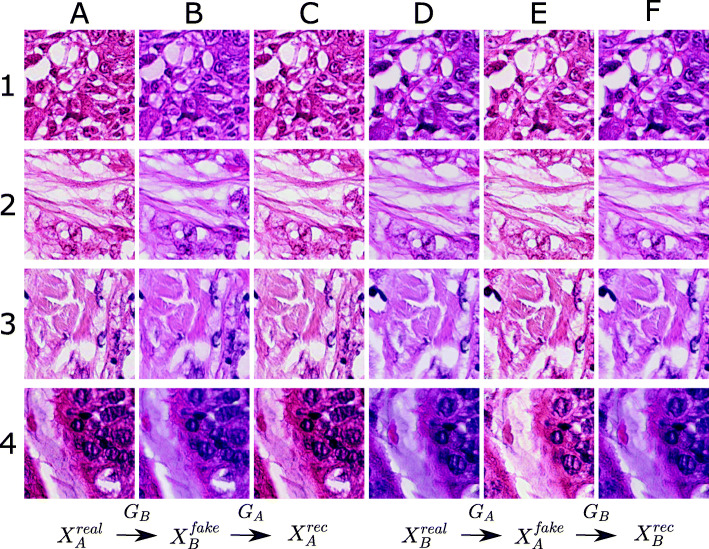
Results gallery from our experiments on the Mitos-Atypia-14 challenge data set. Columns **A-C** refer to the image tiles scanned by the Aperio scanner ($X_{A}^{real}$) being mapped by the generator *G*_*B*_ to produce the corresponding image in the domain of the Hamamatsu scanner ($X_{B}^{fake}$) and the reconstruction from mapping the image back to its source domain ($X_{A}^{rec}$), i.e $X_{A}^{real} \xrightarrow {G_{B}} X_{B}^{fake} \xrightarrow {G_{A}} X_{A}^{rec} $. The same process is done in the reverse direction for image tiles scanned in domain *B*, i.e $X_{B}^{real} \xrightarrow {G_{A}} X_{A}^{fake} \xrightarrow {G_{B}} X_{B}^{rec} $ (column **D-F**). Each row **1-4** presents another example tissue section

Example results of on the Mitos-Atypia-14 data set are shown in Fig. 3. Columns **A-C** refer to the image tiles scanned by the Aperio scanner ($X_{A}^{real}$) being mapped by the generator *G*_*B*_ to produce the corresponding image in the domain of the Hamamatsu scanner ($X_{B}^{fake}$) and the reconstruction from mapping the image back to its original domain ($X_{A}^{rec}$). The same process is done in the reverse direction for image tiles scanned in domain *B* being mapped to domain *A* and backward (columns **D-F**). Each row **1-4** presents another example image.

#### HE-Staining variation

**Fig. 4:**
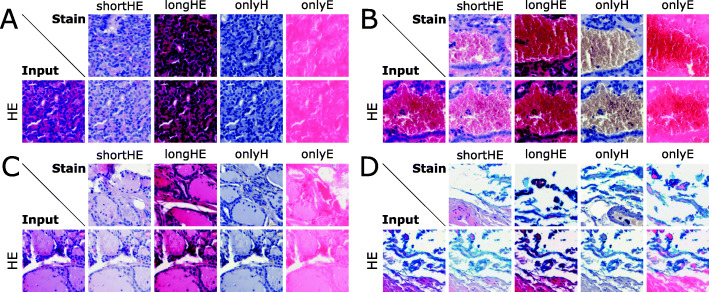
Results gallery from our experiments on the HEV data set for the mapping $G_{B}: X_{A}^{real} \rightarrow X_{B}^{fake} $. Here, the input image is from domain *A* of the standard stained tissue (*HE*) being mapped to domain *B* corresponding to the image-sets *shortHE*, *longHE*, *onlyH*, *onlyE*. Each block **A-D** shows another example tissue section. The top row of each block represents an exemplary image tile of the stain to be mapped into, while the bottom row depicts the input image and the corresponding output for each stain

Figure 4 presents several test results when mapping a standard stained HE-image $X_{A}^{real}$ to one of the four stains of domain $X_{B}^{fake}$. Each block **A-D** shows another example tissue section. The top row of each block represents an exemplary image tile of the stain to be mapped into (*shortHE, longHE, onlyH, onlyE*), while the bottom row depicts the input image (*HE*) and the corresponding output for each stain.

### CycleGAN evaluation

**Fig. 5:**
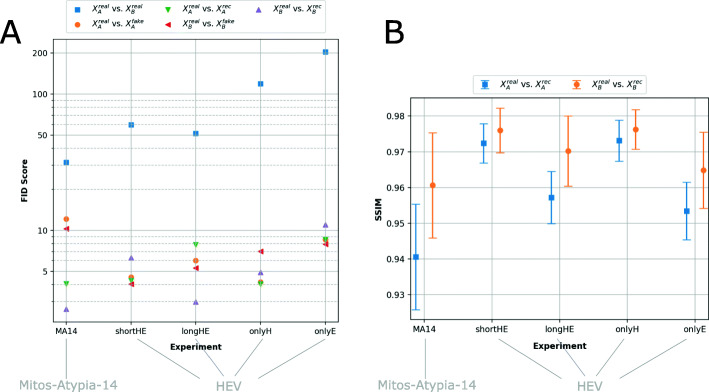
Evaluation of our experiments using FID and SSIM scores. **A**) FID scores between real and generated (fake, rec) images. For identical images the FID is zero, whereas it increases with noise and disturbances. **B**) SSIM scores between real vs. rec images. The SSIM scale ranges from 0 to 1 and is close to zero for hardly similar images. A table with all FID and SSIM scores is presented in the Appendix

For all five experiments FID scores are shown in Fig. 5**A**. As reference, FID scores of all testing images from data sets *A* and *B* (blue) are computed. They range between 31.5 (*MA14*) to 203.68 (*onlyE*). Our experiments achieve on average FID scores for real vs. fake of 7.09 (*A*) and 6.93 (*B*), while for real vs. rec we obtain an average of 5.76 (*A*) and 5.58 (*B*). When mapping images from a source domain to a target domain, the FID scores compared to original images from the target domain improve up to 96% (blue vs. orange, red, green and purple). More precisely, for each experiment it is 76.85*%* (*MA14*), 91.93*%* (*shortHE*), 89.23*%* (*longHE*), 95.76*%* (*onlyH*), 95.57*%* (*onlyE*). A table with all FID is presented in the Appendix.

In addition, SSIM scores (see Fig. 5**B**) are computed between the real and their reconstructed images for each image domain *A* (blue) and *B* (orange). Each value refers to the average SSIM for all test images and the bars represent the corresponding standard deviation (SD). For each set *A* we obtain SSIM scores in the range of *SSIM*=0.94 (*SD*=0.02) (*MA14*) and *SSIM*=0.97 (*SD*=0.01) (*onlyH*), whereas for set *B* we obtain scores between *SSIM*=0.96 (*SD*=0.02) (*MA14*) and *SSIM*=0.98 (*SD*=0.01) (*onlyH*). A table with all SSIM scores is presented in the Appendix.

### Stain normalization in image classification

In order to sow the impact of stain normalization on a downstream task such as image classification or segmentation, a ResNet [19] for the Camelyon16 data set, and its normalized versions, was trained. It should be noted that a standard color normalization to the mean and standard deviation as well as data augmentation with the albumentations package [27] is carried out in the standard training setting. The model learns to classify the images in lymph node parts with (hereinafter called tumor) and without (hereinafter called normal) carcinoma infiltration. Our three ResNets are tested on the Camelyon16 data set (*n*=830 images without and *n*=898 images with tumor) in original, normalized to the HEV data set and normalized to the TL data set as well as to the TL data set (*n*=902 images without and *n*=900 images with tumor) in original, normalized to the HEV data set and normalized to the Camelyon16 data set. As shown in Table 1 all ResNet models work best with images of the same HE-staining from the same training data set (Camelyon16 original, normalized to the HEV or TL data set). Furthermore, the models can best be applied to or transferred to the images from the TL data set, which in turn have the same HE-staining or stain normalization. However, with a best kappa-values of 0.55, this transfer from the Camelyon16 data set to the TumorLymphnode data set does not work very well. Also the ResNet-model trained on hematoxylin normalized images is able to classify other hematoxylin images correctly (kappa-value 0.77 for the Camelyon16 data set and 0.54 for the TL data set). The complete table of the results is shown in the Appendix.

**Table 1:** Kappa-values for the ResNet models that were trained on different versions of the Camelyon16 data set

model / data	*Cam_ori*	*Cam_HE*	*Cam_TL*	*TL_ori*	*TL_HE*	*TL_Cam*
ResNet_ori	**0.85**	0.00	0.12	0.00	0.00	**0.54**
ResNet_HE	0.02	**0.79**	0.28	0.13	**0.55**	0.01
ResNet_TL	0.36	0.10	**0.79**	**0.55**	0.08	0.24

The training images (from the Camelyon16 data set) are 1) original (Cam_ori), 2) normalized by the CycleGAN to the HEV data set (Cam_HE) or 3) the TL data set (Cam_TL), respectively. For each training sets, a ResNet model was trained: 1) ResNet_ori, 2) ResNet_HE and 3) ResNet_TL. All models were tested on images from the Camelyon16 data set (*n*=1728 images) and the TL data set (*n*=1802 images). There were again three versions of both test data sets: one original version (Cam_ori and TL_ori), one version normalized to the HEV data set (Cam_HE and TL_HE), and one version normalized to the Camelyon16 (TL_Cam) or the TL data set (Cam_TL). The best kappa value obtained for each test set (column-wise) on all models is shown in bold

**Table 2:** Overview of our stain normalization experiments

Data set	Experiment Name	Set *A*	Set *B*
Mistos-Atypia-14	*MA14*	Aperio scanner	Hamamatsu scanner
HEV	*shortHE*	standard HE stained	shortened staining time
	*longHE*		prolonged staining time
	*onlyH*		only stained with hematoxylin
	*onlyE*		only stained with eosin
Camelyon16	*Cam_HE*	Camelyon16	standard HE stained
	*Cam_TL*		TumorLymphnode
TumorLymphnode	*TL_HE*	TumorLymphnode	standard HE stained
	*TL_Cam*		Camelyon16

## Discussion

This paper underlines that CycleGANs for image normalization in the context of digital pathology [12] yield a clear clinical benefit. The trained CycleGAN models show compelling results, both visually (Figs. 3 and 4) and quantitatively (Fig. 5) by obtaining FID scores up to 96% better for images mapped to a target domain. The trained models are able to fully convert to the desired color scheme while preserving the structural contents of the original image due to the cycle consistency constraint leading to SSIM scores greater than 0.9 when mapping generated images back to their source domain.

Furthermore, we could confirm that this normalization approach can be beneficial for image classification tasks. Similar to the results of Shaban et al. [10] for the Camelyon16 data set, Yuan et al. [28] for a number of publicly available data sets or de Bel et al. [12] in the application of image segmentation. Compared to earlier approaches on public data sets, in our work we demonstrate how stain normalization improves the classification accuracy, when applied to multiple internal data on a pre-trained model. When normalizing HE-stain images from our institute to the Camelyon16 data set and testing a ResNet-model that is only trained on Camelyon16 data the kappa-value increased more than 50%. Furthermore, we were able to show in a first approach that such a normalization could also enable the use of different colored data sets for downstream tasks, e.g. by converting both data sets to a different staining. However, further work, e.g using more different staining methods, is certainly necessary here.

Some limitations of the used CycleGAN-model can also be seen when mapping images obtained by different scanning devices with varying resolutions. This can cause a loss in structural information despite the consistently good quality of the color normalization. With the HEV data set, the generated images look very realistic compared to the original images in a target domain without any decline in the image content (see Fig. 4).

The CycleGAN approach used here is able to successfully learn every mapping between two image stains and can instantly normalize any unseen image if it is within one of the trained stains. For each other staining, the network needs to be retrained from scratch. However, the network is able to learn even from a small amount of images (1,000-10,000 per set) which can be obtained from a single WSI. In addition, the images do not have to be labeled or paired to learn the mapping between two domains. The network can learn to add a stain to images which is not present in the source domain, e.g we are able to create a full HE-stained image from an image which has only a single stain (*onlyH*, *onlyE*) or vice versa. This can simplify the manual staining process. How this affects other stains besides HE needs further investigation.

## Conclusion

In this paper we show that CycleGANs are a powerful tool for normalization of different variants of HE-stains and tissue types. We validated this approach on data sets covering images from different scanning devices, staining protocols and tissue types and additionally showed the impact on stain normalization on the downstream task of image classification. The method has been successfully applied to compensate for variances resulting from image acquisition as well as from tissue staining while preserving structural content of the images. In order to make use of this approach in a clinical manner, the training process should be accelerated, i.e. using transfer learning, an increased batch size and specialized hardware. The method may be added to various image processing frameworks at WSI level to be applied to tasks such as classification or segmentation.

## Appendix

**Table 3:** FID scores for all experiments between real and generated (fake, rec) images for *A* and *B*

FID	*MA14*	*shortHE*	*longHE*	*onlyH*	*onlyE*
$X_{A}^{real}$ vs. $X_{B}^{real}$	31.5017	59.4240	51.4460	119.0061	203.6761
$X_{A}^{real}$ vs. $X_{A}^{fake}$	12.1464	4.5465	6.0007	4.1793	8.5647
$X_{A}^{real}$ vs. $X_{A}^{rec}$	4.0544	4.2877	7.8630	4.0363	8.5685
$X_{B}^{real}$ vs. $X_{B}^{fake}$	10.3222	4.0365	5.3136	7.0321	7.9218
$X_{B}^{real}$ vs. $X_{B}^{rec}$	2.6451	6.3173	2.9931	4.9206	11.0160

**Table 4:** SSIM scores (SD = standard deviation) for all experiments between real and rec images for *A* and *B*

SSIM (SD)	*MA14*	*shortHE*	*longHE*	*onlyH*	*onlyE*
$X_{A}^{real}$ vs. $X_{A}^{rec}$	0.9406 (0.0147)	0.9724 (0.0055)	0.9572 (0.0073)	0.9731 (0.0057)	0.9534 (0.0080)
$X_{B}^{real}$ vs. $X_{B}^{rec}$	0.9606 (0.0148)	0.9760 (0.0063)	0.9702 (0.0098)	0.9763 (0.0056)	0.9648 (0.0107)

**Table 5:** Kappa-values for the ResNet models that were trained on different versions of the Camelyon16 data set

model / data	*Cam_ori*	*Cam_HE*	*Cam_onlyH*	*Cam_TL*	*TL_ori*	*TL_onlyH*	*TL_HE*	*TL_Cam*
ResNet_ori	**0.85**	0.00	0.00	0.12	0.00	0.00	0.00	**0.54**
ResNet_HE	0.02	**0.79**	0.01	0.28	0.13	0.00	**0.55**	0.01
ResNet_onlyH	0.00	0.00	**0.77**	0.14	0.03	**0.53**	0.00	0.00
ResNet_TL	0.36	0.10	0.53	**0.79**	**0.55**	0.38	0.08	0.24

The best kappa value obtained for each test set (column-wise) on all models is shown in bold

## Data Availability

The code is publicly available at https://github.com/m4ln/stainTransfer_CycleGAN_pytorch. The data set supporting the solutions is available at 10.11588/data/8LKEZF.

## Abbreviations

HE: Hematoxylin-eosin
GAN: Generative adversarial network
FID: Fréchet inception distance
SSIM: Structural similarity index measure
